# Cell organisation, sulphur metabolism and ion transport-related genes are differentially expressed in *Paracoccidioides brasiliensis *mycelium and yeast cells

**DOI:** 10.1186/1471-2164-7-208

**Published:** 2006-08-14

**Authors:** Rosângela V Andrade, Hugo C Paes, André M Nicola, Maria José A de Carvalho, Ana Lúcia Fachin, Renato S Cardoso, Simoneide S Silva, Larissa Fernandes, Silvana P Silva, Eduardo A Donadi, Elza T Sakamoto-Hojo, Geraldo AS Passos, Célia MA Soares, Marcelo M Brígido, Maria Sueli S Felipe

**Affiliations:** 1 Depto. de Biologia Celular, Universidade de Brasília, 70910–900. Brasília-DF, Brazil; 2 Depto de Genética, Faculdade de Medicina de Ribeirão Preto, Universidade de São Paulo, 14040–900, Ribeirão Preto, SP, Brazil; 3 Depto de Bioquímica e Biologia Molecular, Universidade Federal de Goiás, 74001–970, Goiânia, GO, Brazil

## Abstract

**Background:**

Mycelium-to-yeast transition in the human host is essential for pathogenicity by the fungus *Paracoccidioides brasiliensis *and both cell types are therefore critical to the establishment of paracoccidioidomycosis (PCM), a systemic mycosis endemic to Latin America. The infected population is of about 10 million individuals, 2% of whom will eventually develop the disease. Previously, transcriptome analysis of mycelium and yeast cells resulted in the assembly of 6,022 sequence groups. Gene expression analysis, using both *in silico *EST subtraction and cDNA microarray, revealed genes that were differential to yeast or mycelium, and we discussed those involved in sugar metabolism. To advance our understanding of molecular mechanisms of dimorphic transition, we performed an extended analysis of gene expression profiles using the methods mentioned above.

**Results:**

In this work, continuous data mining revealed 66 new differentially expressed sequences that were MIPS(Munich Information Center for Protein Sequences)-categorised according to the cellular process in which they are presumably involved. Two well represented classes were chosen for further analysis: (i) control of cell organisation – cell wall, membrane and cytoskeleton, whose representatives were *hex *(encoding for a hexagonal peroxisome protein), *bgl *(encoding for a 1,3-β-glucosidase) in mycelium cells; and *ags *(an α-1,3-glucan synthase), *cda *(a chitin deacetylase) and *vrp *(a verprolin) in yeast cells; (ii) ion metabolism and transport – two genes putatively implicated in ion transport were confirmed to be highly expressed in mycelium cells – *isc *and *ktp*, respectively an iron-sulphur cluster-like protein and a cation transporter; and a putative P-type cation pump (*pct*) in yeast. Also, several enzymes from the cysteine *de novo *biosynthesis pathway were shown to be up regulated in the yeast form, including ATP sulphurylase, APS kinase and also PAPS reductase.

**Conclusion:**

Taken together, these data show that several genes involved in cell organisation and ion metabolism/transport are expressed differentially along dimorphic transition. Hyper expression in yeast of the enzymes of sulphur metabolism reinforced that this metabolic pathway could be important for this process. Understanding these changes by functional analysis of such genes may lead to a better understanding of the infective process, thus providing new targets and strategies to control PCM.

## Background

The availability of great amounts of raw genomic and transcriptome data collected from several organisms has prompted the development of large-scale gene expression analysis which will ultimately help to unravel the function of many genes in diverse biological contexts. Different approaches such as cDNA microarrays [1-3], *in silico *ESTs subtraction [4,5] and serial analysis of gene expression – SAGE [6,7] are widely employed to assess differential gene expression patterns leading to the discovery of a great number of genes that are over or under expressed in each physiological context. The successful use of the cDNA microarray approach in fungal pathogens such as *Candida albicans *[8-13], *Histoplasma capsulatum *[14] and *Cryptococcus neoformans *[15] has resulted in the identification of genes involved in cell viability and opened new experimental perspectives to understand host-parasite interactions and thus develop new therapeutic approaches to systemic mycoses [8,11].

Paracoccidioidomycosis (PCM) is a human illness endemic to Latin America [16]; its area of incidence spreads non-uniformly from Mexico to Argentina [17], being higher in Brazil, Venezuela, Colombia and Argentina [18,19,16]. An estimation for Brazil points to an incidence rate between 1 and 3 and a mortality rate of 1.4 per million [20]. McEwen *et al*. [21] reported an overall infected population of 10 million individuals in Latin America, 2% of whom will eventually develop the disease. In nature, another important mammalian host is the armadillo *Dasypus novemcinctus *[22]. PCM affects the skin, lymph nodes and various internal organs, including the lungs – where it causes granulomatous processes – and the central nervous system [19,23]. Its clinical presentations range from a localised and benign disease to a progressive and potentially lethal systemic infection [24]. The disease is more frequent in adult males, who account for up to 90% of all cases. Healthy rural workers are the main targets, but PCM affects immunosuppressed individuals as well [25,26], including as much as 30% of AIDS patients [27]. All patients from whom the fungus is isolated must be treated and, in spite of new antifungal drugs, pulmonary fibrosis is still the most frequent sequel. The outcome of infection depends on several factors, including host responses and the virulence of the infecting isolate.

The causative agent of PCM, the thermo-regulated dimorphic fungus *P. brasiliensis*, is believed to be a free-living mycelium saprobe that undergoes transition to the yeast pathogenic form upon temperature change from the environmental 24–26°C to the mammalian body temperature of 37°C. This switch is necessary and sufficient to trigger morphotype interconversion *in vitro*, which makes this fungus an interesting model to study fungal cell differentiation at the molecular level. The biochemical events regulating dimorphic transition in *P. brasiliensis *are yet poorly defined, although relevant molecular-level information on this process has been partially described in the transcriptome analyses of two different *P. brasiliensis *isolates [28-30].

The exact ecological niche of this pathogen is still unknown [17], but *P. brasiliensis *can be retrieved from the soil. The fungus *Penicillium marneffei *is greatly similar in that it is a human opportunistic pathogen that also undergoes thermally-controlled dimorphic transition upon infection, can also infect a wild mammal (the bamboo rat) and has an yet unknown natural reservoir. Genomic data provided evidence that, in the case of *P. marneffei*, the fungus may have a sexual stage as a free-living organism [31].

Phylogenetic analysis of members of the order Onygenales demonstrated a close relationship of *P. brasiliensis *with the pathogenic fungi *Blastomyces dermatitidis, Emmonsia parva *and *Histoplasma capsulatum *[32]. *P. brasiliensis *can be fitted with *B. dermatitidis *and *E. parva *in the family Onygenacea [33]. Recently it was reported that *P. brasiliensis *is in fact a complex of at least three closely correlated phylogenetic species [34]. So far, the sexual phase of the ascomycete *P. brasiliensis *was not reported limiting our knowledge about the mechanisms that contribute to its dimorphism, pathogenicity, and virulence. *P. brasiliensis *isolates shows chromosomal polymorphism; it contains 4–5 chromosomal DNA molecules with molecular sizes ranging from 2–10 Mb [35,36]. The genome size was estimated to be around 30 Mb [37] and DNA sequencing of ~ 50 Kb revealed a density of one gene per 3.5–4.5 Kb, suggesting a total of 7,500–9,000 genes [38].

Recently, our group analysed the transcriptome of the Pb01 isolate, represented by a set of 6,022 clusters. The 16 genes that were then found to be differentially expressed by both methods used – *in silico *EST subtraction and cDNA microarray – were categorised by function. We chose to discuss in that work those that were involved in core metabolic pathways such as sugar metabolism [28]. Now, continued overlap analysis from raw data revealed 66 new genes that are differentially expressed in one or other morphotype. Upon categorisation by known databases we have selected two MIPS [39] classes, which were chosen to be confirmed by northern blotting. Here we present the result of this extended analysis, and discuss the putative roles the differential genes – related to cell organisation and ion metabolism and transport – play in the corresponding morphotype of this pathogen. One of the discussed pathways – *de novo *cysteine synthesis from inorganic sulphate, a branch of sulphur metabolism – was almost entirely up-regulated in the yeast form. The importance of sulphur metabolism to the life cycle of pathogenic fungi has been extensively reviewed elsewhere [40,41] and recently new data from microarray experiments have arisen from work in *H. capsulatum *that support a role of organic sulphate in the maintenance of the yeast phase [14]. In a previous report [42], the importance of organic sulphates to the growth and differentiation of *P. brasiliensis *was assessed. This phenomenon demanded further investigation and prompted us to assess up- and downregulation of sulphur metabolism genes in mycelium and yeast cells and also dimorphic transition in both directions without inorganic sulphate as a sulphur source. We have thus found that this compound is unnecessary for the process.

## Results and discussion

### Differentially expressed genes identified by *in silico *EST subtraction and cDNA microarray

Comparative gene expression profiling in dimorphic fungi can reveal key proteins involved in commitment to differentiation and gene regulation. From the 66 new PbAESTs (*P. brasiliensis *assembled expressed sequence tags) identified in this work, thirteen of which correspond to up-regulated genes in mycelium and fifty four which are differential for yeast cells (Tables 1 and 2). This set complements the one generated previously [28], which included 16 genes that were differential by the same overlap analysis and also 30 genes that were differential according to *in silico *EST subtraction alone. MIPS functional categories [43] were used to classify the 66 PbAESTs into 14 major groups (data not shown). Gene categorisation revealed some that are involved in energy production (11%) – this was expected considering the adaptation process that is required for the mycelium-to-yeast transition; control of cell wall organisation (10%); ion metabolism and transport (8%); transcription, translation and ribosome structure (8%); virulence and oxidative stress (4%). Manual annotation under stringent criteria of sequence alignment with other dimorphic fungi gene sets allowed us to ascribe a putative biological function to many of those genes. The genes that belonged in two categories – cell wall organisation and ion metabolism and transport – were selected for confirmation by northern blotting.

**Table 1:** Mycelium up-regulated genes identified by *in silico *ESTs subtraction and cDNA microarray.

PbAEST	Acession Numbers (GenBank)	Annotated function	Number of reads		P-value^*a*^	Fold change	Accession Number/Best-hit organism/E-value	Functional categories
							
			M	Y				
202	CA582032	1,3-beta-glucosidase*	7	2	0.036942	12.3	AAL09828.1/*C. immitis*/1.0E-132	Control of cell organization: Cell wall and membrane
2155	CA582352	Peroxisomal membrane protein PEX16 (peroxin-16)	7	0	0.004174	1.4	EAL88469.1/*A. fumigatus*/3.0E-64	
186	CA583085	HEX*	13	8	0.049272	3.4	EAL91716.1/*A. fumigatus*/3.0E-66	

2496	CA583518	Iron-sulphur cluster nifU-like protein*	5	1	0.048854	1.7	EAL90111.1/*A. fumigatus*/8.0E-58	Ion transport
4179	CN245816	Potassium transporter protein*	0	1	-^*b*^	5.2	CAA08814.1/*N. crassa*/4.0E-22	

1420	CN247275	U1 small nuclear ribonucleoprotein	9	1	0.00526	1.6	EAL91268.1/*A. fumigatus*/1.0E-60	Transcription

1029	CA582332	Methyltransferase	32	1	0.000000	2.1	EAL84975.1/*A. fumigatus*/1.0E-56	Others
2096	CA581148	Unkown	20	1	0.000006	5.6	-	
514	CA583322	Unkown	15	1	0.000138	23.4	-	
1045	CA581951	Unkown	13	2	0.001769	24	-	
1178	CN247241	Unkown	10	0	0.000535	8.5	-	
1664	CN247289	Unkown	10	3	0.018648	2.5	-	

^*a *^FDR = 4,8% and Q-value < 5%.

^*b *^Not significant by Audic-Claverie's method.

* Up-regulated genes confirmed by northern blotting.

** Not assayed by cDNA microarray but confirmed as up-regulated in mycelium by northern blotting.

**Table 2:** Yeast up-regulated genes identified by *in silico *ESTs subtraction and cDNA microarray.

PbAEST	Acession Numbers (GenBank)	Annotated function	Number of reads		P-value^*a*^	Fold change	Accession Number/Best-hit organism/E-value	Functional categories
							
			M	Y				
1422	CA581980	Alpha-1,2-mannosyltransferase (Alg11)	4	11	0.019803	2.0	EAL88400.1/*A. fumigatus*/1.0E-130	Control of cellular organization: Cell wall and membrane
4988	CN253911	Alpha 1,3-glucan synthase*	-	1	-	5.7	AAV52833.1/*P brasiliensis*/4.0E-93	
2162	CN238153	Putative WW domain protein (probable membrane protein)	4	12	0.013092	3.6	EAL85876.1/*A. fumigatus*/6.0E-17	
136	CA582283	Involved in cytoskeletal organization and cellular growth (verprolin)*	4	10	0.029289	4.0	NP_013441.1/*S. cerevisiae*/2.3	

667	CA583397	Adenylylsulphate kinase	3	8	0.038949	2.1	EAL90409.1/*A. fumigatus*/1.0E-88	Ion transport and metabolism
48	CA582091	ATP-sulphurylase	10	18	0.023038	4.8	EAL92915.1/*A. fumigatus*/0.0	
2031	CA581274	Outer mitochondrial membrane protein porin	1	14	0.000207	1.3	XP_323644.1/*N. crassa*/1.0E-108	
2724	CA581633	P-type Cu(2+) transporting ATPase*	0	1	-^*b*^	3.8	NP_009854.1/*S. cerevisiae*/1.7E-20	

635	CN247312	ATP citrate lyase	1	7	0.014984	1.9	EAL88915.1/*A. fumigatus*/0.0	Energy
2016	CN242578	ATPase inhibitor; Inh1	2	14	0.000835	2.7	NP_010100.1/*S. cerevisiae*/4.0E-08	
563	CA583982	Cytochrome c oxidase subunit VII	11	43	0.000002	2.1	AAT77147.1/*P. brasiliensis*/3.0E-26	
2398	CN240705	Disulfide isomerase	3	8	0.038949	2.1	EAL91387.1/*A.fumigatus*/3.0E-61	
540	CN240558	Cytochrome C oxidase biogenesis protein	0	5	0.015111	1.8	XP_214182.2/*R. norvegicus*/1.0E-06	

578	CA582837	Pyruvate dehydrogenase e1 component beta subunit	2	7	0.033994	1.6	EAL86696.1/*A. fumigatus*/2.0E-99	
407	CA583387	Succinyl-CoA synthetase alpha subunit	7	19	0.004468	2.6	EAL91981.1/*A. fumigatus*/1.0E-155	
284	CN239025	Ubiquinol-cytochrome C reductase complex ubiquinonE-binding protein QP-C	0	4	0.030475	1.5	EAL90680.1/*A. fumigatus*/7.0E-29	

378	CA580847	Argininosuccinate synthase	0	6	0.007492	1.7	NP_229577.1/*T. maritime*/4.0E-77	Amino acid metabolism and transport
1618	CA583639	Aromatic-L-amino-acid decarboxylase	1	33	0.00000	17.2	EAL86509.1/*A. fumigatus*/0.0	
125	CA583825	Glycine cleavage system h protein	4	9	0.042192	1.4	EAL90537.1/*A. fumigatus*/6.0E-36	

1674	CA583874	Aldolase	5	14	0.010368	37.9	AAL34519.2/*P. brasiliensis*/0.0	C-compound and carbohydrate metabolism
42	CA581699	Phosphoglycerate kinase	1	10	0.002512	2.6	EAL90363.1/*A. fumigatus*/0.0	

9	CA581893	Beta-ketoacyl synthase (Cem 1)	1	5	0.045709	2.2	EAL87667.1/*A. fumigatus*/1.0E-88	Lipid, fatty-acid and isoprenoid metabolism
780	CA581145	GPR/FUN34 family protein	0	11	0.000225	14.9	EAL87502.1/*A. fumigatus*/6.0E-67	
1989	CA581550	Acetyl-coenzyme A synthetase (AcetatE – CoA ligase) (Acyl-activating enzyme)	1	9	0.004605	2.0	EAL89682.1/*A. fumigatus*/0.0	
1550	CA582818	NADH-cytochrome b5 reductase	0	6	0.007492	5.4	EAL88164.1/*A. fumigatus*/1.0E-86	

300	CA581937	Nucleoside diphosphate kinase	6	58	0.00000	1.6	AAP85295.1/*A. fumigatus*/2.0E-67	Nucleotide metabolism

547	CA583473	6,7-dimethyl-8-ribityllumazine synthase	0	6	0.007492	1.4	AAD55372.1/*A. fumigatus*/9.0E-56	Metabolism of vitamins, cofactors, and prosthetic groups
924	CN240624	Coproporphyrinogen III oxidase	2	7	0.033994	2.7	EAL88456.1/*A. fumigatus*/0.0	
867	CA580742	NADH pyrophosphatase	1	5	0.045709	5.7	EAL85969.1/*A. fumigatus*/1.0E-159	
1490	CA583063	Pyridoxamine 5'-phosphate oxidase	0	10	0.000453	3.5	AAC28862.1/*S. commune*/2.0E-32	
447	CA580589	NADH:ubiquinone oxidoreductase B18 subunit	1	10	0.002512	1.4	EAL92195.1/*A. fumigatus*/9.0E-33	

488	CA582788	Exonuclease II	1	5	0.045709	1.9	EAL85993.1/*A. fumigatus*/1.0E-138	Transcription, translation and ribosome structure
165	CN241393	RNP domain protein	3	13	0.003962	1.8	EAL89070.1/*A. fumigatus*/5.0E-81	
2436	CA580512	Splicing factor u2af 35 kd subunit	2	7	0.033994	2.5	EAL86523.1/*A. fumigatus*/1.0E-103	
253	CN240426	Zinc finger, C3HC4 type	0	5	0.015111	1.4	NP_593329.1/*S. cerevisiae*/3.0E-10	
551	CN239696	Ribosomal protein L35**	5	10	0.044755	-	AAL08563.1/*P. brasiliensis*/1.0E-63	
979	CA582579	60S ribosomal protein L7/L12 precursor	1	8	0.008358	1.3	EAL89813.1/*A. fumigatus*/4.0E-49	

175	CA581863	Complex I intermediatE-associated protein CIA30 precursor	4	15	0.003399	5.6	EAL92946.1/*A. fumigatus*/1.0E-114	Protein fate and Secretion
832	CN242383	Glutathione S transferase	1	7	0.014984	2.0	NP_588171.1/*S. pombe*/7.0E-42	
2387	CA584103	Non-classical export protein (Nce1)	1	7	0.014984	55.6	EAL87256.1/*A. fumigatus*/1.0E-29	
1823	CA583903	Profilin	1	5	0.045709	1.3	NP_014765.1/*S. cerevisiae*/8.0E-14	

4188	CN245872	Mating type protein (MAT1–2)*	1	0	-	8.0	EAL89707.1/*A. fumigatus*/2.0E-36	Mating Type

50	CA581392	Cu-Zn superoxide dismutasE-related*	0	8	0.001842	2.1	CAB97297.1/*N. crassa*/3.0E-30	Virulence and oxidative stress

2059	CN241260	Ribosome associated protein (Stm1)	6	31	0.000007	1.7	EAL92489.1/*A. fumigatus*/2.0E-32	Others
2005	CA580764	Signal peptide protein	1	6	0.026442	2.3	EAL93249.1/*A. fumigatus*/7.0E-68	
39	CA581046	Unknown	0	6	0.007492	2.2	-	
33	CA582496	Unknown	0	8	0.001842	3.1	-	
1442	CA581846	Unknown	3	16	0.000836	4.5	-	
2399	CA581839	Unknown	1	5	0.045709	2.5	-	
512	CA583749	Unknown	0	6	0.007492	4.3	-	
639	CA581506	Unknown	0	7	0.003715	1.7	-	
718	CN247671	Unknown	0	6	0.007492	1.8	-	
765	CA581478	Unknown	0	10	0.000453	3.9	-	
529	CA580398	Unknown	1	5	0.045709	18.8	-	

^*a *^FDR = 4,8% and Q-value < 5%.

^*b *^Not significant by Audic-Claverie's method.

* Up-regulated genes confirmed by northern blotting.

** Not assayed by cDNA microarray but confirmed as up-regulated in yeast by northern blotting.

### Mycelium and yeast up-regulated genes involved in cell organisation

**Figure 1:**
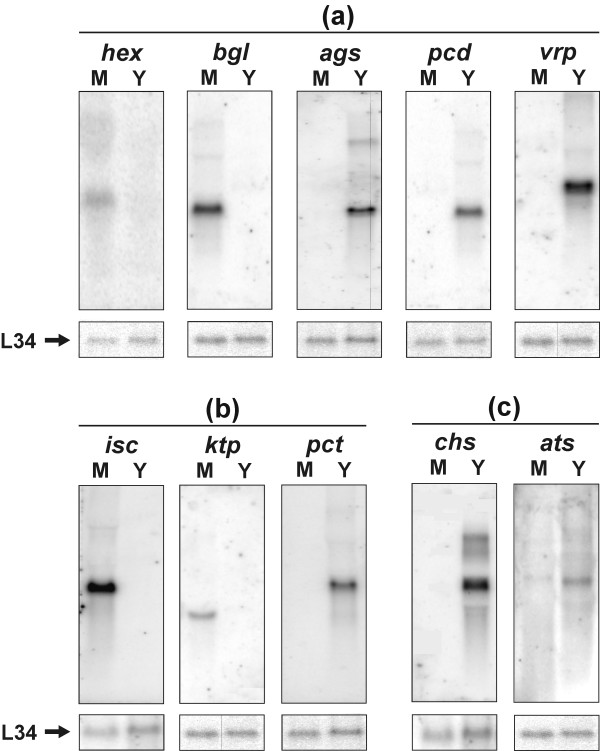
**Northern blot analysis of mycelium and yeastup-regulated genes of *P. brasiliensis***. Total RNA samples from both mycelium (M) and yeast (Y) were blotted onto nylon membranes and hybridised against gene-specific radiolabelled probes: **(a) **Control of cell organisation: *hex *– Hexagonal peroxisome protein, *bgl *–1,3 beta-glucosidase, *ags *– alpha 1,3-glucan synthase, *cda *– Chitin deacetylase, *vrp *– Verprolin; **(b) **Ion transporters: *isc *–Iron-sulphur cluster-like protein, *ktp *– Potassium transporter, *pct *– Putative P-type Cu(2+) transporting ATPase; **(c) **Sulphur metabolism: *chs *– Choline sulphatase, *ats *– ATP sulphurylase. The constitutive 60S ribosomal protein L34 was used as a loading control.

The *hex *and *bgl *genes, which code for the hexagonal peroxisome protein and 1,3 β-glucosidase, respectively, were up-regulated in mycelium cells and are categorised as involved in cell wall, membrane and cytoskeleton organisation (Figure 1a). The hexagonal peroxisome protein has been identified in different filamentous ascomycetes such as the plant pathogen *Magnaporthe grisea *[44] and in *Neurospora crassa *[45], being the major protein of the Woronin body, a septal pore-associated organelle [46,47]. HEX1p has been shown to seal septal pores in response to cellular damage and is strongly implicated in cell integrity maintenance [45]. In *M. grisea*, *hex1 *mutants present delayed host penetration and subsequent disruption of invasive hyphal growth in plants. Inability of these mutants to survive under nitrogen starvation *in vitro *has also been observed [44]. Deletion of *hex1 *in *N. crassa *eliminates Woronin bodies from the cytoplasm and results in hyphae that exhibit a cytoplasmic-bleeding morphotype in response to cell lysis [45]. It was thus proposed that the Woronin body represents a new category of peroxisome acting in the maintenance of cellular integrity and virulence in filamentous fungi [45]. We hypothesise that these highly specialised vesicles are involved in the protection of *P. brasiliensis *against cellular damage as well as its survival during invasive growth and host colonisation in the process of infection. Future investigations are required to elucidate the role of Woronin bodies/HEX1 protein in *P. brasiliensis*.

Another mycelium up-regulated gene codes for β-1,3-glucosidase, an enzyme that hydrolyses the O-glycosidic linkages of β-glucan. This polysaccharide is an important cell wall constituent in *P. brasiliensis *mycelium cells in contrast with α-glucans, which predominate in the yeast cell wall [48]. A hypothesis formulated by Kanetsuna *et al*. [49] and modified by San Blas and San Blas [50] explains the differentiation from mycelium to yeast and vice-versa based on a change on cell wall composition. At 37°C, there is an increased synthesis of chitin and α-glucan, and low levels of β-glucan, which results in the yeast form. In contrast, at 22°C, α-glucan synthesis occurs at low rates and long β-glucan fibrils are formed in the budding spots. In keeping with these morphological and biochemical events, 1,3-β-glucosidase increased levels are correlated to the shift to the mycelium phase.

Other three genes coding for proteins from the same category were confirmed to be up-regulated in yeast cells: *ags *(α-1,3-glucan synthase), *cda *(chitin deacetylase) and *vrp*-verprolin (Fig. 1a). The *P. brasiliensis *1,3-α-glucan synthase gene was first described by Pereira *et al*. [51]. Recently, it was demonstrated that it is strongly up-regulated in yeast cells [28,52], which was confirmed in this work by northern blotting analysis. Rappleye *et al*. [53] silenced the 1,3-α-glucan synthase gene in *H. capsulatum *and demonstrated that α-(1,3)-glucan is an important virulence factor and affects the ability of *H. capsulatum *to kill macrophages and colonise murine lungs. In *C. neoformans*, mutants for 1,3-α-glucan synthase failed to assemble the capsule, which is an important virulence factor of this pathogen [54]. Morphogenetic transition is the essence of *P. brasiliensis *life cycle: for instance, low levels of α-1,3-glucan in the cell wall of the yeast form have been correlated with low virulence [55]. Virulent cultures of *P. brasiliensis *isolates grown i *n vitro *for long periods have thinner cell walls, low α-1,3-glucan levels and are consequently less virulent [56]. Our results suggest that α-glucan synthase is involved in the dimorphic transition of *P. brasiliensis *and possibly in its virulence. The cell wall is an essential and dynamic fungal structure that has been implicated in several pathogenic processes. Being absent in mammalian cells, it may be a relevant target to drug therapies. In this context, the gene that encodes α-1,3-glucan synthase was demonstrated to be a virulence factor using RNAi approaches in *Cryptococcus neoformans *[54] and *H. capsulatum *[53], and seems to be an ideal target for new antifungal drugs. In *P. brasiliensis *glucan polymers constitute 95% of yeast cell wall [49] and thus any interference in cell wall synthesis through glucan synthases is likely to affect virulence directly.

Chitin deacetylase enzyme (CDA) catalyses the conversion of chitin to chitosan by deacetylation of N-acetyl-D-glucosamine residues. Chitosan is a flexible, soluble polymer that integrates the cell wall of some fungi, such as *S. cerevisiae *[57] and *C. neoformans *[58]. In *S. cerevisiae*, chitosan is only found during sporulation [59]. The molecular characterisation of two sporulation-specific chitin deacetylase genes, *CDA1 *and *CDA2*, both of which contribute to spore wall rigidity, was described previously [59]. In *S. cerevisiae*, *cda1 *mutants present a more diffuse chitosan layer, while their surface layer remains intact. In *cda2 *mutant cells, by comparison, the chitosan layer is not detected at all. In the spore walls of *cda1 *and *cda2 *mutants both outer layers are missing due to defects on wall maturation. However, in *C. neoformans*, a study reported that chitin is present in the yeast cell wall and most of it is continually deacetylated to chitosan. Mutants for chitin deacetylase show suppression of growth due to the lack of chitosan and therefore have a reduced infection capability [58]. The same study hypothesized that this constant remodelling of the cell wall contributed to cellular integrity in this fungus. In *P. brasiliensis*, we identified a highly expressed *cda *gene in yeast cells that presents similarity to the *C. neoformans*. If the *C. neoformans *model is closer to what is found in *P. brasiliensis*, then chitin synthase and chitin deacetylase may be potential targets to antifungal therapy.

Verprolin is required for a fully polarised distribution of cortical actin patches and viability at high temperature. This is the first time that verprolin is described in *P. brasiliensis*, a pathogen that has as an intrinsic characteristic the ability to grow at the human body temperature, 37°C. The inability of *vrp-1 *mutants to grow at 37°C was reported by Naqvi *et al*. [60] in the non-pathogenic yeast *S. cerevisiae*. Likewise, we hypothesise that verprolin is involved in the ability of *P. brasiliensis *to grow at 37°C and in cell cytoskeleton organisation since this gene is over expressed in yeast cells. Considering that the actin cytoskeleton plays a crucial role on fundamental processes such as cell growth, differentiation and migration, localised membrane growth, endocytosis, and cell division [61], this protein is likely to play a key role in cell maintenance and viability of *P. brasiliensis *inside the host cell.

### Mycelium and yeast up-regulated genes involved in ion metabolism and transport

Two genes putatively implicated in ion transport were confirmed to be highly expressed in mycelium cells: *isc *and *ktp*, an iron-sulphur cluster protein and a cation transporter, respectively. In contrast, a putative P-type cation pump (*pct*) was up-regulated in the yeast form (Figure 1b).

It has been reported that the ISC protein is responsible for mitochondrial uptake of iron and seems to monitor the cytoplasmic levels of this ion. In *S. cerevisiae*, the double knock-out of the homologues *ISU1 *and *ISU2 *is lethal. Defective mutants are distinguished by iron accumulation in the mitochondrial matrix and its respective decrease in the cytosol [62]. In *C. neoformans*, complementation, cloning and sequencing of such genes has recently been accomplished [63]. It has long been hypothesised that iron is a limiting factor for infectivity during cryptococcosis as well as in other systemic mycoses, in that the host normally provides only limited amounts of this compound. Arango and Restrepo [64]demonstrated iron availability to be essential for growth of mycelium and yeast of *P. brasiliensis*; but especially for mycelium, whose growth was totally prevented by the addition of the iron chelator phenanthroline to the medium, an effect observed only to a lesser extent in yeast. The effect of phenanthroline was reversed partially in mycelium and totally in yeast by addition of excess iron. This is in good agreement with the overexpression of the ISC protein in the mycelial phase. In *P. brasiliensis *it could be involved in monitoring the amount of iron in the environment and in providing a means of storage of this metal.

The *ktp *sequence from *P. brasiliensis *aligned best with potassium transporter proteins of the HAK family, which are mainly implicated in the resistance to potassium starvation. In *N. crassa*, the closest homolog of *P. brasiliensis*, KTP coexists with another potassium transporter of the TRK family [65]. It has been hypothesised that soil organisms are universally equipped with a powerful K^+^-concentrating apparatus, as these organisms are faced with a very diluted and variable environment, thus being forced to pump potassium in against a steep gradient [65]. This is likely to be the case of *P. brasiliensis*, whose ecological niche for the mycelium form is thought to be the soil.

Another yeast up-regulated gene is *pct*, a putative member of the E1-E2 (P-type) family of ATPases. These are ATP-dependent proteins which regulate transmembrane flow of all relevant cations, including Na^+^, H^+^, Mg^2+^, Ca^2+^, Cd^2+^, Cu^2+ ^and K^+ ^[66]. In *C. albicans*, the E1-E2 ATPase gene, *CDR1*, confers resistance to both copper and silver, the latter being used as an antimicrobial agent [67]. A similar function could be attributed to the *P. brasiliensis pct *gene, although alignment data are insufficient to identify which cation this protein transports.

### Sulphur metabolism

**Figure 2:**
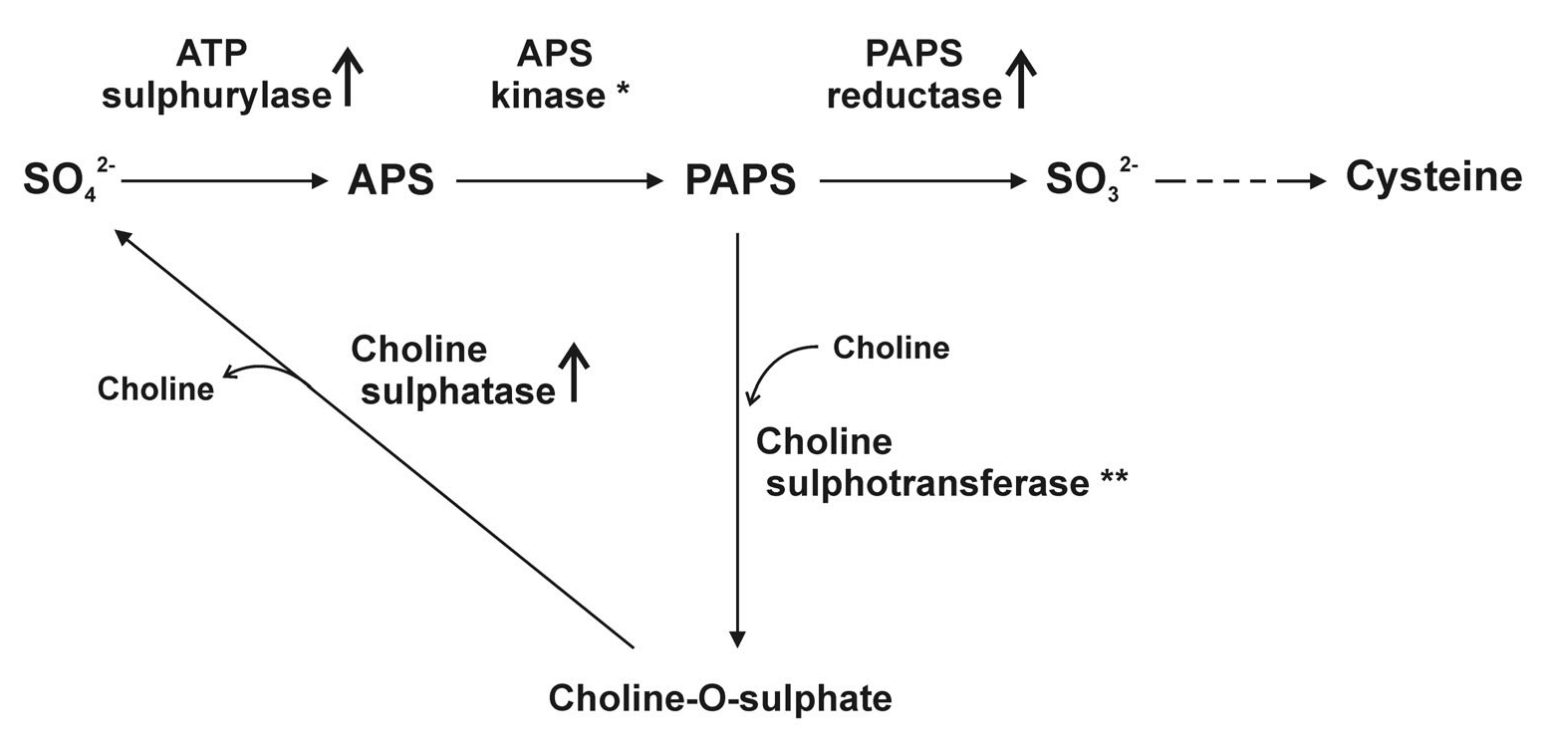
**Up-regulated genes encoding enzymes from the cysteine *de novo *biosynthesis pathway**. Arrows indicate enzymes identified as up-regulated both by *in silico *subtraction, cDNA microarray and confirmed by northern blotting experiments. (*) enzyme identified as up-regulated by both *in silico *subtraction and cDNAs microarray but not assayed by northern blotting. (**) indicates an enzyme not found in the transcriptome of *P. brasiliensis*.

Several enzymes from the cysteine *de novo *biosynthesis pathway (Figure 2) were shown to be up-regulated in the yeast form of *P. brasiliensis*. Our previous analysis [28] had already confirmed over expression of paps reductase (the third in the pathway). *In silico *EST subtraction and cDNA microarray showed yeast up-regulation for atp sulphurylase and aps kinase; the former was confirmed by northern blotting (Figure 1c). Thus, we can strongly suggest that the yeast form synthesises cysteine actively from inorganic sulphate.

**Figure 3:**
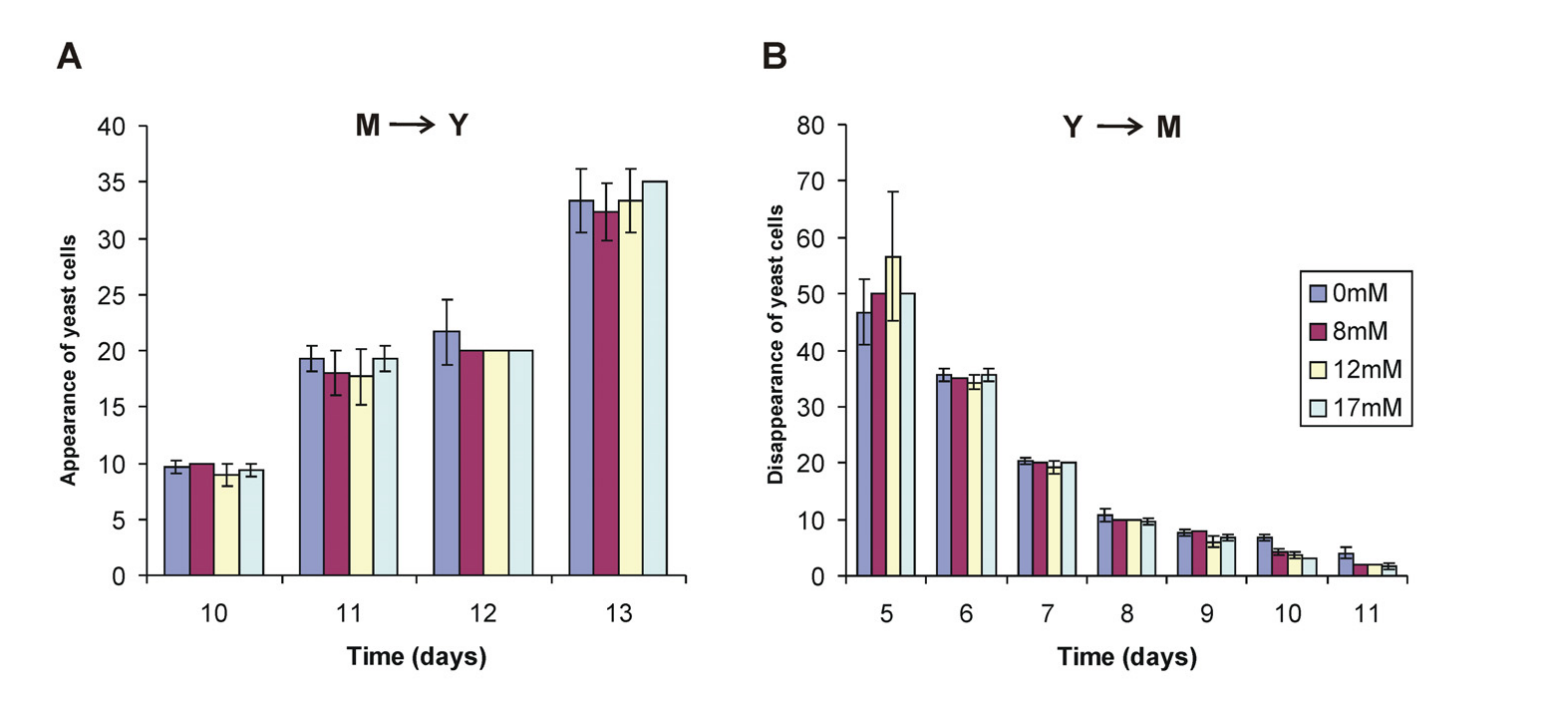
**Cell differentiation of *P. brasiliensis *in modified MVM medium without inorganic sulphate**. The fungus was grown in four different concentrations of sulphate salts (0, 8, 12 and 17 mM; the latter is the original concentration of MVM medium). (A) The appearance of yeast cells was verified daily in the transition from mycelium to yeast after temperature shift to 37°C, (B) The disappearance of yeast cells was verified daily in the transition from yeast to mycelium after temperature shift to 22°C. Triple samples were counted for each time point. The coloured boxes indicate the average of the three samples and bars represent the standard deviation of the mean. As observed, the presence or absence of inorganic sulphate did not affect transition in either direction.

In order to reinforce these data, we have evaluated the importance of inorganic sulphate to growth and differentiation. Auxotrophy of *P. brasiliensis *yeast for several sources of organic sulphate – including cysteine itself and sulphydrylic compounds – has been reported before [42]. It was concluded then that organic sulphate deprivation suppressed growth in the yeast phase and prevented mycelium-to-yeast differentiation, whereas the mycelial phase is able to grow on either inorganic or organic sulphur [68]. Also, the saprophytic, mycelial form of *H. capsulatum *is prototrophic while the pathogenic yeast form requires cysteine [69]. It has been reported that exogenous cysteine is required for both yeast phase growth and morphological transition from mycelium-to-yeast of *H. capsulatum *[41,70]. In this work, both mycelium and yeast cells of *P. brasiliensis *were incubated in modified MVM medium without inorganic sulphate, apart from the negligible amounts present in the trace elements solution. Dimorphic transition was assessed in the mycelium to yeast direction and in the opposite way.Sustained growth was observed for both morphotypes (data not shown) and, upon the corresponding temperature shifts, differentiation was successfully triggered in both directions (Figure 3). Thus, inorganic sulphate seems to be unnecessary for the transition, quite contrarily to organic sulphate. In this context, it is interesting to consider a branch of the cysteine biosynthetic pathway (Fig. 2). In fungi and plants a fraction of PAPS, which is toxic to fungi if it reaches high cytosolic levels, is used by choline sulphotransferase to produce choline-O-sulphate [40], which serves as an osmoprotectant and cytosolic sulphur store in these organisms. We have not found a homologue of choline sulphotranferase in *P. brasiliensis *to date, but the enzyme choline sulphatase, which degrades its product to choline and sulphate, is also over expressed in the yeast morphotype, as confirmed here (Figure 1c) and previously reported [52]. The *C. neoformans met3 *mutant, which lacks ATP sulphurylase activity, had a substantial defect in melanin formation, significantly reducedgrowth rate, and greatly increased thermotolerance. In the murine inhalation infection model, the *met3 *mutant was avirulent and was deficient in its ability to survive in mice [71]. In this context, disrupting the genes encoding choline sulphatase or ATP sulphurylase in *P. brasiliensis *should reveal its role in the growth, maintenance of yeast cells and pathogenicity of this fungus. It is interesting that another intracellular pathogen of humans, the bacterium *Mycobacterium tuberculosis*, depends on sulphur compounds for expression of its full virulence, drug resistance and overall survival inside the macrophage. It has developed a very efficient sulphate activation pathway (SAC) that ensures constant synthesis of PAPS at high rates, from which sulphate may be distributed to other synthetic pathways [72]. The SAC includes the bacterial counterparts of ATP sulphurylase and APS kinase, the latter of which performs PAPS synthesis by coupling it with GTP hydrolysis by a GTPase that is also present in SAC. Whether similar mechanisms are present in pathogenic fungi such as *P. brasiliensis *remains to be investigated.

## Conclusion

**Figure 4:**
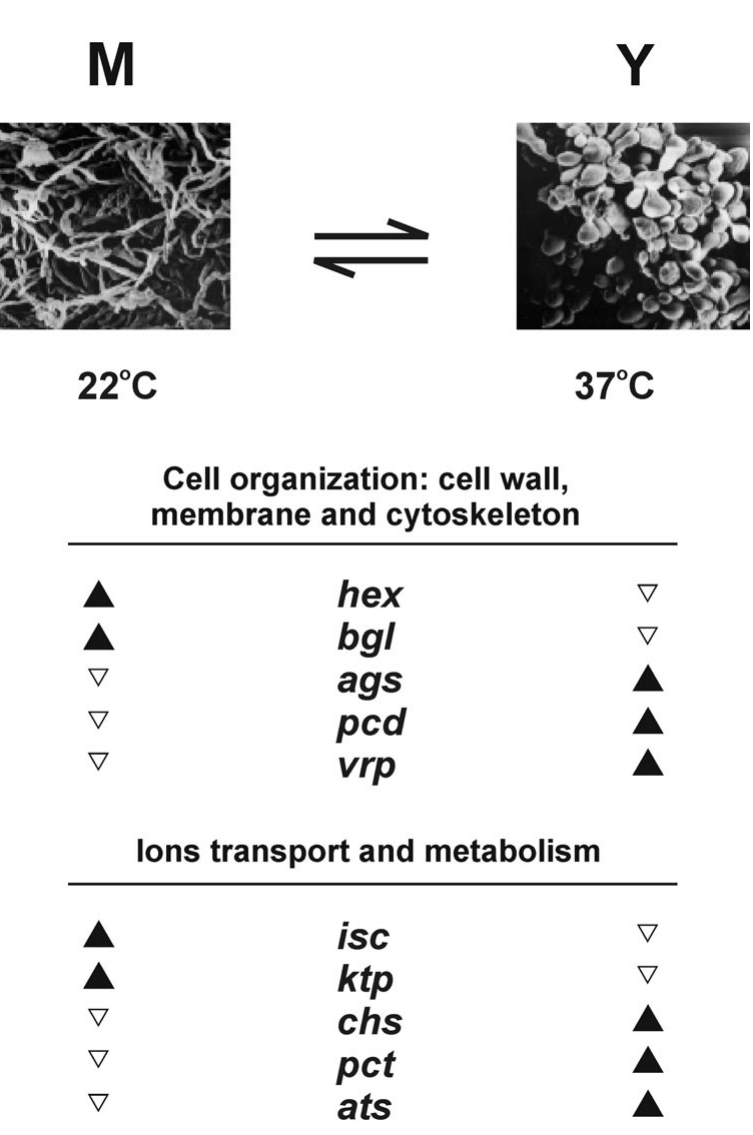
**Genes involved in cell organisation (cell wall, membrane and cytoskeleton), sulphur metabolism and ion transport**. Genes that were identified as up-regulated in mycelium (22°C) or yeast (36°C) cells of *P. brasiliensis *are represented by black arrowheads. Electron microscopy was performed by Silva *et al*. [78].

Taken together, these data show that several genes involved in cell organisation and ion metabolism/transport are differential in their expression along dimorphic transition, which is in accordance with the proposed model for this process in Figure 4. While α-glucan is synthesised during yeast phase due to high expression of 1,3 α-glucan synthase, β-glucan is degraded by the action of 1,3 β-glucosidase during hyphal growth. The *cda *gene is probably involved in the cell wall synthesis of yeast cells, since it is over expressed in this phase. In addition, genes related to septal sealing and cytoskeleton organisation (*hex *and *vpr*) are also probably implicated in the stabilisation and maintenance of mycelium and yeast cells in the environment and at 37°C in the human host. Also, the differential expression pattern of genes that control metabolism and transport of potassium, iron, copper and sulphur ions suggests that they may influence directly the pathogen adaptation to the host environment. Specifically, in spite of the undisturbed growth and differentiation on depletion of inorganic sulphate, the over expression of enzymes from *de novo *cysteine synthesis lends support to previous findings about the importance of this pathway to yeast metabolism. Understanding these changes by functional analysis of such genes may lead to a better understanding of the infective process, thus providing new targets and strategies to control PCM.

## Methods

### Strains and cultures

*P. brasiliensis *clinical isolate Pb01 (ATCC-MYA-826) was used in this study. Cells from both mycelium and yeast forms were grown on semi-solid Fava Neto's medium [73] for 7 to 10 days at 22°C or 37°C, respectively.

### Overlap analysis – *in silico *EST subtraction and cDNA microarrays

This work was based on the output of previous large-scale expression analysis experiments, as outlined in reference 28. Briefly, we have constructed a λZAP II^® ^(Invitrogen) cDNA library from mycelium and yeast mRNA fractions and 5'-sequenced the mass-excised cloned fragments with the T7 vector primer. Raw sequence data were quality-assessed by PHRED and assembled by CAP3, thus generating a set of 6,022 PbAESTs (singlets and contigs). For functional annotation of sequences we used the nr (NCBI) database. *In silico *electronic subtraction was performed according to the Audic and Claverie [74] statistical approach, with a 95% confidence rate. For cDNA microarray 1,152 clones were selected and PCR-amplified for spotting onto nylon-membranes in triple experiments. Hybridisation against [α-^33^P] dCTP-labeled total RNA from mycelium or yeast and phosphor imager signal capture were performed as in [28]. After signal quantification and background subtraction [75], statistical analysis was carried out with the SAM (Significance Analysis of Microarrays) method [76]. Data from both experiments were overlapped to identify differential genes, thus generating the set of 66 sequences we used in this work.

### Northern blot analysis

Total RNA (15μg) from mycelium and yeast cells of *P. brasiliensis *was separated on denaturing 1,5 % agarose gel and blotted onto a Hybond-N membrane (GE Healthcare). Probes were radiolabeled using [α-P^32^]dATP by random priming according to supplier's instructions (Invitrogen), purified and used in overnight hybridisation (50% formamide, 4X SSPE, 5X Denhardt's solution, 0,1% SDS, 100μg/ml herring sperm DNA) at 42°C. The membranes were then washed under stringency conditions of 2X SSPE-0.1% SDS at 65°C for 1h. Signal bands were visualised using the Typhoon 9210 Phosphor Imager (GE HealthCare).

### Dimorphic transition without inorganic sulphate

We incubated both mycelium and yeast cells on modified versions of McVeigh and Morton's medium – MVM [77] where ammonium and magnesium sulphate salts were gradually replaced by their chloride counterparts, in the following chloride concentration set points: 0, 8, 12 and 17 mM, where the first corresponds to the original recipe and the last, to virtual absence of inorganic sulphate, apart from negligible amounts in the trace elements solution (~ 8 μM). Molar concentrations of both magnesium and ammonium were thus conserved. We have also evaluated whether dimorphic transition occurred normally in the medium without inorganic sulphur. To achieve this, five flasks containing 100 ml of modified MVM were inoculated with comparable amounts of mycelium (100 mg wet mass) and yeast (2.5 × 10^7 ^cells) previously grown on standard MVM. Samples were incubated in rotating shakers (120 rpm) at 36 and 22°C, respectively, thus triggering dimorphic transition. Fungal viability and progress of transition were assessed by serial 100 μl sampling every 24 hours (three independent samples). Each sample was coloured with Janus Green and the number of yeast cells was counted in a light microscope with the aid of a Neubauer counting chamber.

### Accession numbers

The accession numbers of the EST sequences analysed in this work are shown in the Tables 1 and 2.

## Abbreviations

*ags *alpha 1,3-glucan synthase

*aps *adenosine 5'-phosphosulphate

*ats *ATP sulphurylase

*bgl *1,3 beta-glucosidase,

BLAST basic local alignment search tool

*cda *chitin deacetylase

cDNA complementary DNA

*chs *choline sulphatase

COG clusters of orthologous groups

*e-value *extreme value distributionESTs

ESTs expressed sequence tags

GO gene ontology

*hex *hexagonal peroxisome protein

*isc *iron-sulphur cluster-like protein

*ktp *potassium transporter

MIPS Munich information center for proteins sequences

PAPS phosphoadenylyl-sulfate reductase

PbAETs *P. brasiliensis *assembled EST sequences

PCM paracoccidioidomycosis

*pct *putative P-type Cu(2+) transporting ATPase

SAGE serial analysis of gene expression

SAM significance analysis of microarrays

*vrp *verprolin

## Authors' contributions

RA and MF planned and designed the study, developed the experiments and the data analysis, wrote the main draft of the paper and support the preparation of the figures and tables. HP supports the discussion of the results and revised the manuscript. AN participated in the *in silico *ESTs subtraction analysis of the raw data generated by the transcriptome project. MC analysed the results of the microarray experiments, helped in the manuscript edition, and prepared the figures. AL executed the microarray experiments. MC, RC and MB participated in the normalization process of the microarray raw data and helped to make the statistical analyses. SS participated of the differentiation experiment involved of the inorganic sulphur, and of the preparation of the RNA of *P. brasiliensis *to make the microarray experiments. LF participated of the analysis of the cell wall organization. SP helped in the ESTs amplification and on the analysis of the sulphur metabolism. GP, ES, ED designed the microarray experiments. CS participated on the Pb ESTs annotation. All authors read and approved the final manuscript.
